# Intermittent Cold Exposure Enhances Fat Accumulation in Mice

**DOI:** 10.1371/journal.pone.0096432

**Published:** 2014-05-02

**Authors:** Hyung sun Yoo, Liping Qiao, Chris Bosco, Lok-Hei Leong, Nikki Lytle, Gen-Sheng Feng, Nai-Wen Chi, Jianhua Shao

**Affiliations:** 1 Department of Pediatrics, University of California San Diego, La Jolla, California, United States of America; 2 Department of Pathology, University of California San Diego, La Jolla, California, United States of America; 3 Veterans Affairs San Diego Healthcare system, and Department of Medicine, University of California San Diego, La Jolla, California, United States of America; Graduate School of Medicine, the University of Tokyo, Japan

## Abstract

Due to its high energy consuming characteristics, brown adipose tissue (BAT) has been suggested as a key player in energy metabolism. Cold exposure is a physiological activator of BAT. Intermittent cold exposure (ICE), unlike persistent exposure, is clinically feasible. The main objective of this study was to investigate whether ICE reduces adiposity in C57BL/6 mice. Surprisingly, we found that ICE actually increased adiposity despite enhancing Ucp1 expression in BAT and inducing beige adipocytes in subcutaneous white adipose tissue. ICE did not alter basal systemic insulin sensitivity, but it increased liver triglyceride content and secretion rate as well as blood triglyceride levels. Gene profiling further demonstrated that ICE, despite suppressing lipogenic gene expression in white adipose tissue and liver during cold exposure, enhanced lipogenesis between the exposure periods. Together, our results indicate that despite enhancing BAT recruitment, ICE in mice increases fat accumulation by stimulating de novo lipogenesis.

## Introduction

Obesity is a metabolic disease characterized by overexpansion of white adipose tissue (WAT). Although genetic predisposition is important in the development of obesity, chronic positive energy balance has been considered the main cause of obesity in the general population. Therefore, correcting energy imbalance is an ideal therapy for obesity. Unfortunately, commonly used therapeutic approaches such as dieting and exercise are not efficient at containing the obesity epidemic.

WAT and BAT are the two main types of fat in mammals. WAT is the primary energy depot that stores energy as triglyceride-enriched lipid droplets. By contrast, BAT is considered as an energy dispenser that consumes significant amounts of chemical energy toward thermogenesis [1]–[3]. Due to its inconspicuous appearance in adult humans, BAT was previously thought to exist only in infants. Using new technology, recent studies have demonstrated the presence of metabolically active BAT in adults [4]–[8]. Cold temperature stimulates BAT activation and increases energy expenditure [5], [7], [8]. Furthermore, BAT activation is correlated with decreased adiposity in humans [5]–[7], [9]. Therefore, BAT activation has been proposed as a potential new therapeutic approach for obesity.

Cold exposure activates BAT thermogenesis. However, prolonged exposure to cold in humans has been limited by cardiovascular and respiratory complications [10]–[13]. Therefore, repetitive or intermittent cold exposure (ICE) may be a more realistic approach to activate BAT in humans. Although cold exposure and ICE have been used in rodents and even human subjects, their effects on systemic energy metabolism and adiposity are not fully understood. For rodents, many studies reported that cold exposure enhances both fatty acid oxidation and glucose-derived lipogenesis in BAT, but its effects on WAT were controversial [14]–[16]. Furthermore, contradictory effects on body weight and WAT have been observed in both mice and rats [17]–[22]. For humans, although ICE enhances BAT recruitment, its effects on systemic adiposity have been controversial [23], [24]. Therefore, it is necessary to clarify the effect of cold exposure on body fat before applying ICE to treat obesity.

Here, by using C57BL/6 mice, we have investigated whether and how ICE alters adiposity. Similar to human subjects and rats, ICE induced BAT recruitment in mice. Unexpectedly, ICE induced fat accumulation, an effect that cannot be attributed to hyperphagia or stress. Remarkably, ICE induced lipogenic gene expression in both WAT and liver during the non-exposure period. Therefore, our results demonstrate that in spite of inducing BAT recruitment, ICE increases de novo lipogenesis in WAT and liver then enhances fat accumulation in mice.

## Research Design and Methods

### Mice and cold exposure

C57BL/6 mice at 12–16 weeks of age housed under standard conditions with ad libitum access to food were used. They were singly caged for all cold exposure studies to avoid huddling. All cold exposure started at 8:30–9:00 am. For acute cold exposure (ACE), single caged mice were transferred to a 4°C room or a room (23–25°C) next to the cold room to ensure the mice have a similar environment, such as noise and light. The cold room is 20 meters away from the EchoMRI system. After each scanning, which took 1.5 min, the mouse was immediately returned to cold room or the place where control mice located. Therefore, the interruption of cold exposure was reduced to minimal 4–5 min. For ICE, the exposure time to cold air was gradually increased: 3 hours on the first day and an additional hour for each subsequent day up to 6 hours per day. This was followed by a 6-hr cold exposure daily for 8–10 days. For feeding restriction during ICE, each mouse was provided a daily quota equal to its own baseline food intake measured one week prior to the ICE. Tissue samples were collected immediately after cold exposure (ACE) or at 9:00 PM in the ad libitum state (ICE). All animal protocols were approved by the University of California San Diego Institutional Animal Care and Use Committees.

### Body Composition

Body composition was determined by MRI scanning of conscious mice (EchoMRI 100, Houston, TX). Each scan took 1.5–2 minutes. During ACE, body composition was scanned hourly. For ICE, mice were scanned before and after each cold exposure. Interscapular BAT and inguinal and epididymal WAT were weighed at the end of the ICE study.

### Indirect Calorimetry

Energy expenditures were measured using the Oxymax Lab Animal Monitoring System (Columbus Instruments, Columbus, OH). Mice were individually housed in the chamber at room temperature with ad libitum access to food and water and under regular light/dark cycles. Oxygen consumption (VO_2_) was normalized to lean body mass measured just prior to the clorimetry.

### Hepatic triglyceride content and secretion rate

For TG content, frozen liver tissues (approximately 50 mg) were homogenized in 2∶1 chloroform∶methanol to extract triglycerides. After adding NaCl to 0.88%, samples were centrifuged and the aqueous phase decanted. The organic phase was evaporated and then dissolved in isopropranol. Triglyceride levels were measured using a commercial kit (Wako Chemicals, Richmond, VA). Hepatic TG secretion rates were measured after injection (i.v.) of Poloxamer-407 to block LPL as previously described [25].

### Adipocyte morphometry

Sections of fixed fat tissue stained with H&E were imaged at 20× magnification. To size adipocytes using ImageJ software (NIH, Bethesda, MA), the area of measurement was randomly chosen. Adipocytes were outlined to measure the cross-section areas, excluding those that were too small or too large (<100, >1000 arbitrary units) to avoid errors in the outlining process.

### Immunoblotting, quantitative RT-PCR, glucose tolerance and insulin challenge tests

Previously described procedures were used [25].

### Statistical analyses

Data are expressed as mean ± standard error of the mean (SEM). Student t-tests and ANOVA were used to determine p values. P<0.05 was considered statistically significant.

## Results

### ICE induces fat accumulation in mice

**Figure 1:**
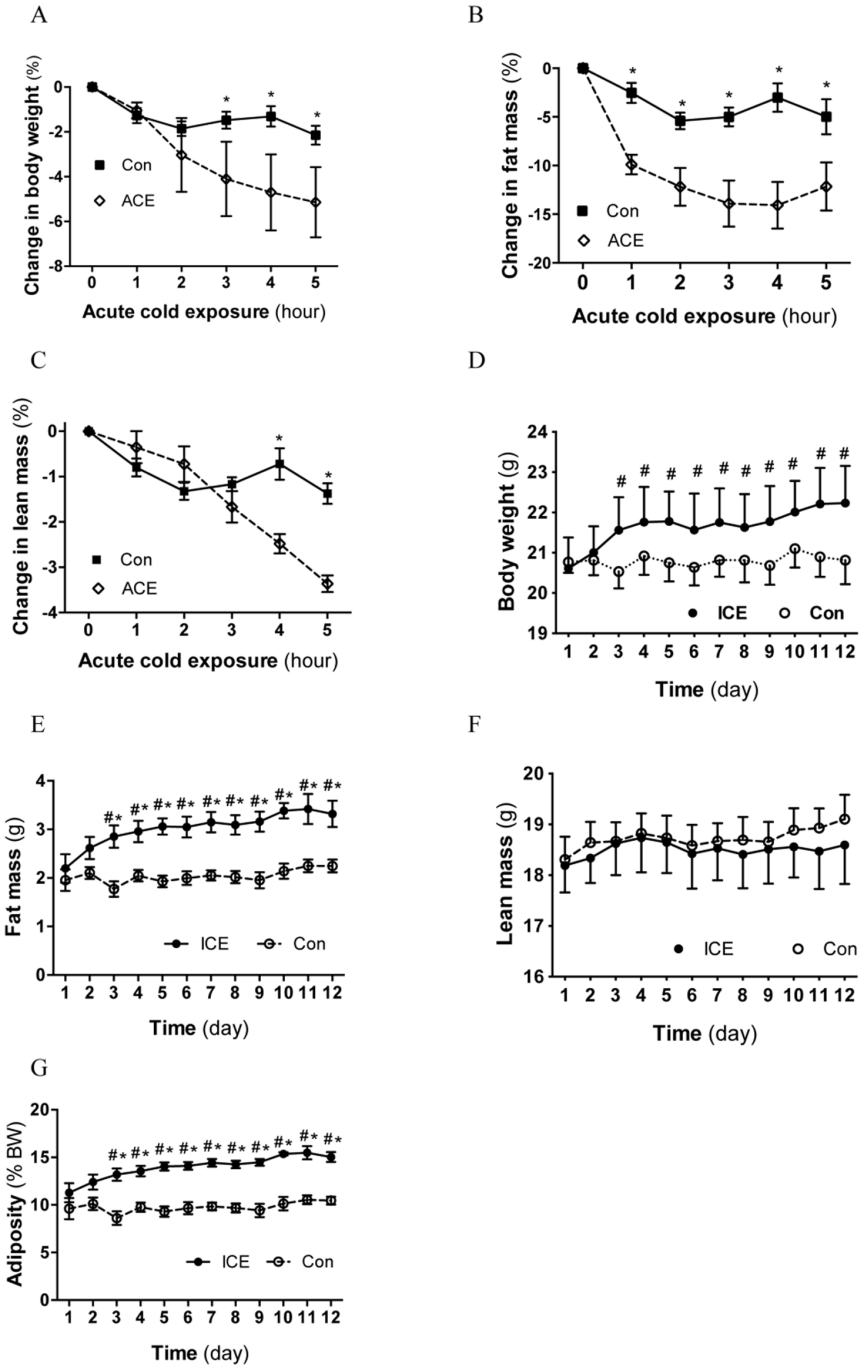
ACE reduces body weight whereas ICE increases body fat accumulation. Singly caged male mice were transferred into cold room (4°C) for cold exposure with ad libitum access to food and water. For ACE study (A–C), body composition was scanned before and hourly during cold exposure. The % decrease in body weight (A), fat mass (B), and lean mass (C) was calculated based on the measurement immediately before the cold exposure. An age-matched cohort was used as controls. For ICE study (D–G), female mice were exposed to cold air (4°C) daily at 9:30 am for 3, 4 and 5 hours on days 1, 2 and 3 respectively, and for 6 hours from days 4 through 12. Body composition was scanned daily just prior to the cold exposure. Adiposity was calculated as fat percentage of body weight (G). Data presented are mean ±SEM, n = 5–6, *p<0.05 Control vs. ICE mice at a given time point; #<0.05 vs. day 1 baseline.

We investigated the effects of both acute and intermittent cold exposures (ACE and ICE) on adiposity. We found that ACE for 5 hours induced progressive loss of body weight, fat mass, and even lean mass (Fig. 1A–C). The reduction of fat mass was significant after 1 hr of ACE and stabilized after 3 hrs (Fig. 1B). The decrease in lean mass was significant after 4 h of cold exposure (Fig. 1C). Interestingly, control mice also showed a slight reduction of body weight and fat mass during the study (Fig. 1A&B), which may represent diurnal fluctuation of body composition or the result of manipulation. Together, these results indicate that ACE enhances catabolism and weight loss in mice.

Similar to ACE, ICE also induced a significant loss of body fat during each cycle of cold exposure (Fig. S1). This loss was recovered prior to the subsequent cycle of cold exposure (Fig. S1). Surprisingly, after two cycles of exposure, body weight and fat mass of ICE mice increased significantly above baseline (Fig. 1D&E, measured right before each cycle of cold exposure). Compare to controls, ICE mice showed trends of increases in body weight (Fig. 1D) and decreases in lean mass (Fig. 1F), but the differences did not reach statistical significance. However, ICE mice demonstrated significant increases in fat mass and adiposity (Fig. 1E&G). Interestingly, within 20 days of stopping ICE, the adiposity of ICE mice decreased to the levels of control mice (data not shown). Together, these results indicate that ICE increases fat accumulation in mice.

### ICE increases BAT recruitment without altering global insulin sensitivity or energy expenditure

**Figure 2:**
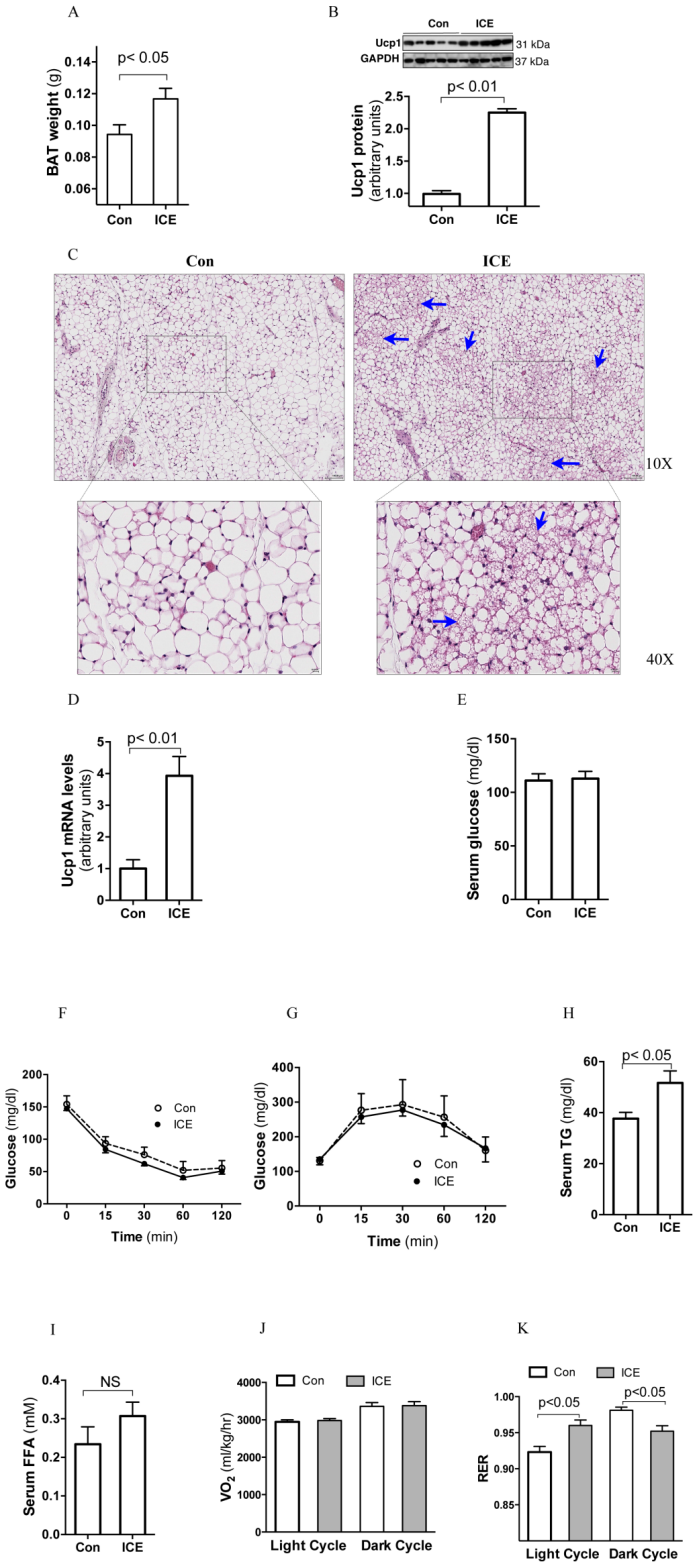
ICE increases BAT recruitment and has no effects on basal energy expenditure or insulin sensitivity. After 12 days of ICE, samples were collected at 9AM after overnight recovery at room temperature in the fed state. Interscapular brown adipose tissue weight (A) and Ucp1 protein levels (B) were compared between Con and ICE mice. (C) Inguinal fat pads were collected for hematoxylin and eosin staining. BAT-like structures or beige cells are marked with blue arrows. (D) Ucp1 mRNA levels in inguinal fat. (E)Basal blood glucose, (H) TG and (I) FFA. F&G, after 12 days of ICE, insulin challenge test and glucose tolerance test were carried out after an overnight fasting at room temperature. J&K, indirect calorimetry was performed on the same male mice before and after ICE treatment. ICE-treated mice were allowed to recover overnight at room temperature. VO_2_ was normalized to lean body mass. Data shown are mean ±SEM, n = 4–6.

Similar to results from cold acclimation studies [26], ICE mice exhibited a significant expansion of interscapular BAT mass and an upregulation of Ucp1 expression in that tissue compared to control mice (Fig. 2A&B). Moreover, their inguinal fat pads contained more beige cells or brown fat-like structure (Fig. 2C) and expressed more Ucp1 mRNA than control (Fig. 2D). These results indicate that ICE treatment increases BAT recruitment.

BAT activation has been suggested to increase energy expenditure and improve glucose and lipid metabolism, especially in the obese state. However, our study showed that ICE did not significantly alter glycemic levels either in the fasting state (Fig. 2E) or after challenge with insulin (Fig. 2F) or glucose (Fig. 2G). These results suggest that ICE does not alter basal systemic insulin sensitivity or glucose homeostasis in mice. Since insulin is important in regulating lipogenesis, which was enhanced in liver and WAT of ICE mice (following sections), the results of glucose and insulin challenge test cannot rule out the improvement of insulin sensitivity in liver and WAT. We also found that ICE led to an increase of serum triglycerides (Fig. 2H) and a trend toward increased free fatty acid levels in serum, albeit not reaching statistical significance (Fig. 2I).

Having shown that ICE increased BAT recruitment and body fat, we then investigated its effect on energy expenditure using indirect calorimetry to compare each mouse before and after ICE treatment. To our surprise, ICE caused no significant alteration in oxygen consumption in the basal state (Fig. 2J). However, the respiratory exchange ratio (RER) was significantly impacted by ICE, being increased during the light cycle and decreased during the dark cycle (Fig. 2K). It should be pointed out that this metabolic study, which was conducted at room temperature after 12 days of ICE treatment, was not designed to address energy expenditure during the cold exposure. Therefore, any transient BAT activation during cold exposure could have escaped detection by the calorimetry.

### Food intake and stress did not play a major role in ICE-induced fat accumulation

**Figure 3:**
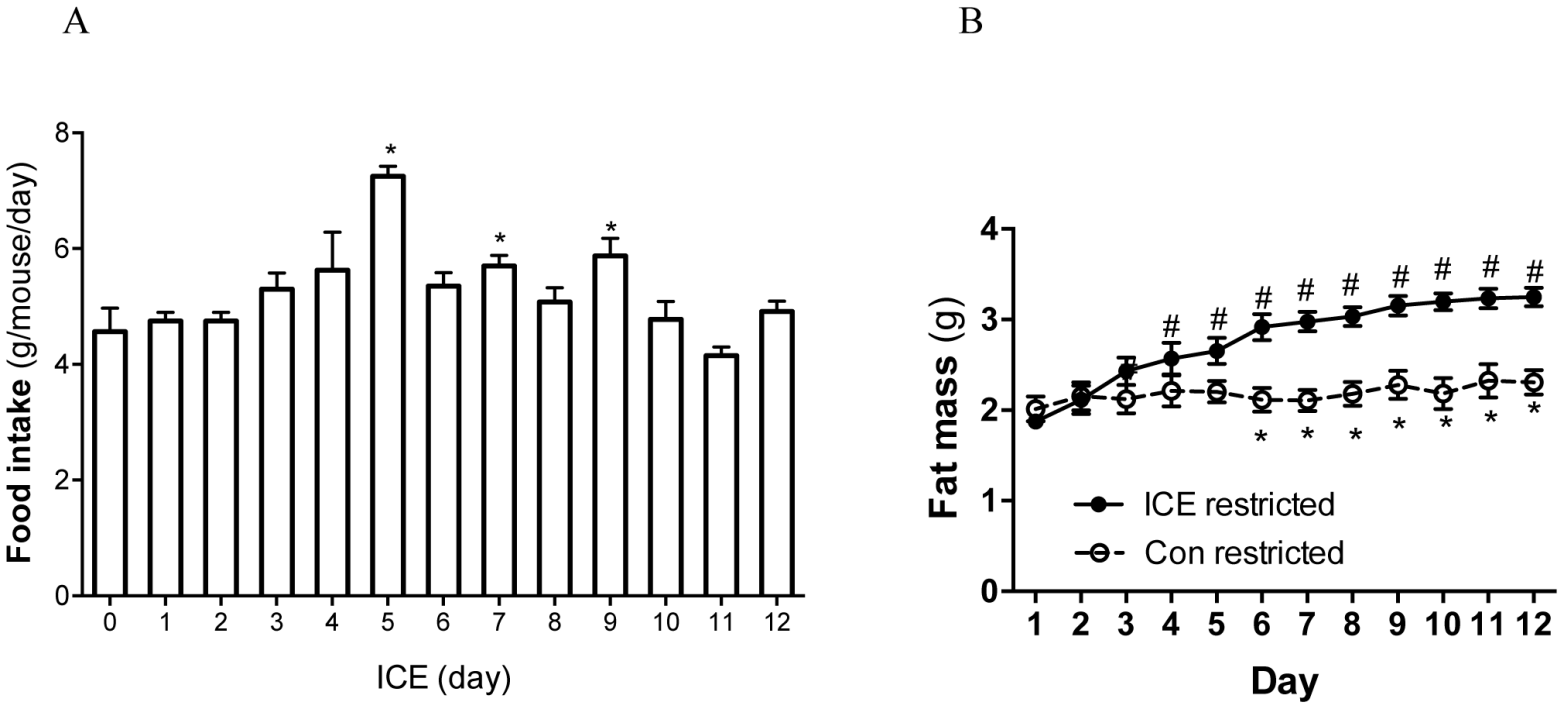
ICE-induced fat accumulation does not require increased food intake. (A) Daily food intake of each mouse was measured during ICE. (B) During ICE, daily food supply was restricted the average amount prior to ICE. Body composition was measured daily before each time of cold exposure. Data presented are mean ±SEM, n = 6, *p<0.05 day 0 (A) or diet-restricted control vs. ICE mice at a given time point (B); # p<0.05 vs. day 1 for the same cohort.

Cold exposure often increases food intake in rodents [27], [28]. We indeed observed hyperphagia during ICE that peaked on day 5 before returning toward baseline by day 12 (Fig. 3A). To determine the role of hyperphagia in ICE-induced fat accumulation, we restricted the food intake of ICE mice to its pre-ICE intake level. Despite this dietary restriction, ICE-induced expansion of fat mass again reached statistical significance within 4 days (Fig. 3B). We therefore concluded that ICE-induced fat accumulation does not depend on hyperphagia.

Cold exposure is a physical stress, and stress has been implicated in the development of obesity [29]. We therefore used physical restraint to simulate stress to better understand ICE-induced fat accumulation. We found that daily 4-hour restraint for 5 days did not lead to increased body fat (data not shown). Since restraint-induced stress might differ from cold-induced stress, this result does not rule out a role of stress in ICE-induced adiposity. It does suggest that stress is probably not a major contributor of ICE-induced fat accumulation in mice.

### ICE increases lipid accumulation and hepatic TG production

**Figure 4:**
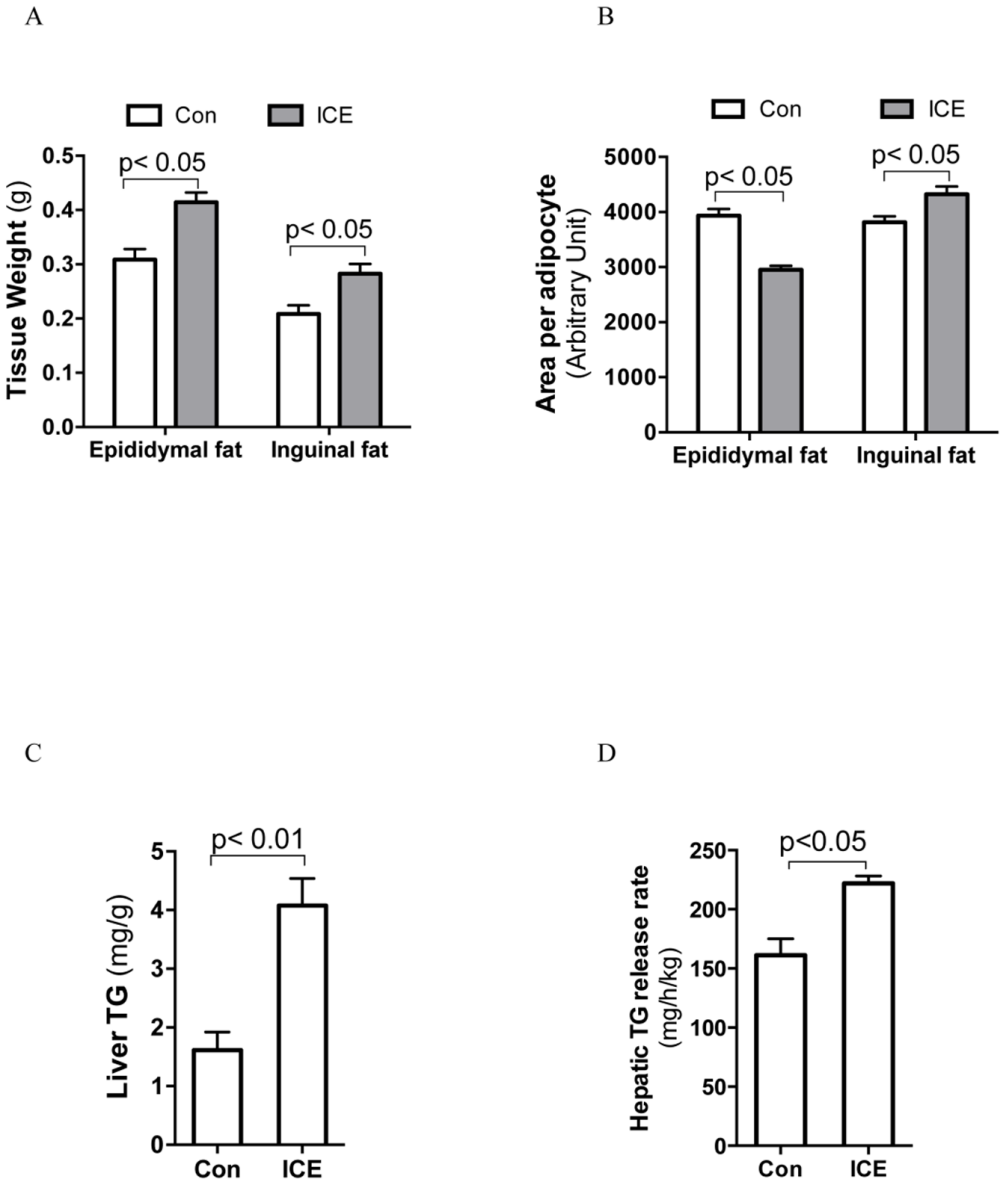
ICE increases WAT mass and hepatic TG production. After 12 days of ICE treatment, inguinal and epididymal fat and liver tissue were collected in the fed state. (A) Tissue weight was measured. (B) Areas of individual adipocytes in H&E-stained sections were quantified using ImageJ software. (C) Liver TG content was normalized to tissue weight. (D) To measure hepatic TG secretion rate, the LPL inhibitor Poloxamer-407 was injected into the tail vein after an overnight fast at room temperature. Blood samples were collected at 0, 15, 30, and 45 min, and TG levels were measured to calculate TG secretion rates as previously described (25). Data presented are mean ±SEM, n = 6–8.

Both epididymal and inguinal fat pads expanded significantly in ICE mice (Fig. 4A). Interestingly, adipocytes (excluding beige cells) became smaller in epididymal fat pads but bigger in inguinal fat pads after ICE (Fig. 4B), suggesting that ICE increases both lipid accumulation and white adipocyte recruitment. Although ICE mice did not exhibit visible hepatic steatosis (Fig. S2), their liver contained more TG and secreted more TG than controls (Fig. 4C&D). These data indicate that ICE increases hepatic TG production.

### ICE increases de novo lipogenesis during the non-exposure period

**Figure 5:**
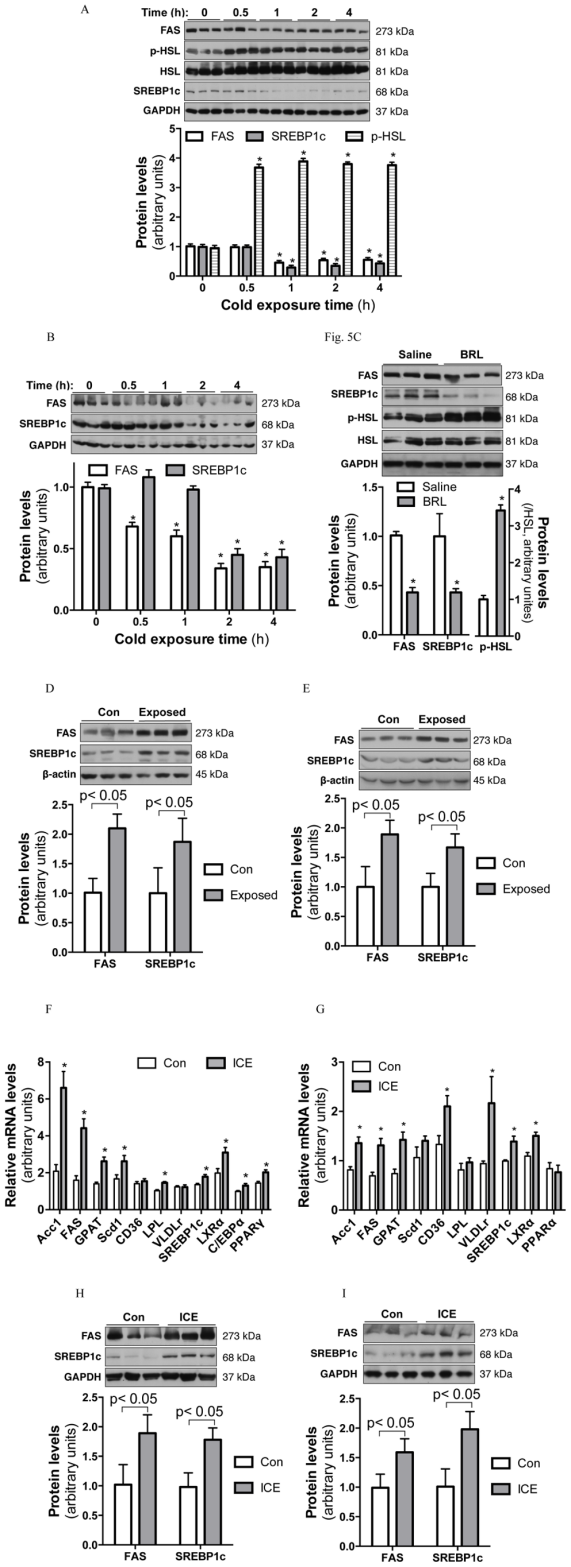
Effects of cold exposure on lipogenic gene expression in WAT and liver. Tissue samples of epididymal fat (A&D) and liver (B&E) were collected during ACE (A&B) or 9 hours after room temperature recovery (D&E). (C) Epididymal fat samples were collected 2 h after BRL i.p. injection (0.2 ug/g body weight). Protein levels were determined by immunoblotting with the indicated antibodies. Levels of Phosphorylation of HSL were divided by total HSL protein and presented as fold increases over basal (A&C). Data presented are mean ±SEM, n = 4–6 mice per time point, *p<0.05 vs. time 0 or Saline controls. F–I, male mice were subjected to ICE for 12 days. Tissue samples of epididymal fat (F&H) and liver (G&I) were collected at 9 PM on day 12 at room temperature without fasting. mRNA (F&G) and protein (H&I) levels were determined by real-time PCR and Western blotting. Data presented are mean ±SEM, n = 8 per group, *p<0.05 vs. control mice.

To investigate the mechanisms underlying ICE-enhanced fat accumulation, we examined the expression and activation status of key regulators of adipogenesis and lipogenesis in WAT and liver. During cold exposure, there were significant increases in the phosphorylation of hormone sensitive lipase (HSL) in both epididymal (Fig. 5A) and inguinal WAT (data not shown), suggesting of increased lipolysis. Protein levels of fatty acid synthase (FAS) and mature SREBP1c progressively decreased in both epididymal fat (Fig. 5A) and liver (Fig. 5B) during cold exposure. Since cold exposure stimulates the sympathetic nervous system (SNS) to increase thermogenesis, we used the β3-specific adrenergic receptor agonist BRL37344 (BRL) to study the effect of acute SNS activation on lipogenesis. We found that BRL injection, like acute cold exposure, reduced FAS and SREBP1c protein levels while increasing HSL phosphorylation in inguinal fat (Fig. 5C). These results suggest that cold exposure stimulates lipolysis while suppressing de novo lipogenesis.

In contrast, 9 hours after recovery from ACE, the expression of FAS and SREBP1c in epididymal fat (Fig. 5D) and liver (Fig. 5E) was significantly higher than in mice without prior cold exposure. These results suggest that cold exposure triggered subsequent rebound de novo lipogeneis in both WAT and liver.

We also compared the expression of lipogenic genes between ICE and control mice under regular housing conditions. We found robustly increased mRNA levels of de novo lipogenic genes, such as FAS, acetyl-CoA carboxylase (ACC1), glycerol-3-phosphate acyltransferase (GPAT), LXRα and SREBP1c, in WAT (Fig. 5F) and liver (Fig. 5G) of ICE mice. Similarly, elevated protein levels of FAS and SREBP1c were observed in epididymal fat and liver (Fig. 5H&I). These results further support the notion that ICE increases de novo lipogenesis during the non-exposure period. In addition, significantly increased LPL expression in WAT as well as CD36 and VLDLr expression in liver suggest that ICE stimulates both tissues to take up lipids from circulating lipoproteins (Fig. 5F&G).

The enlarged epididymal fat mass of ICE mice and their smaller adipocyte size imply that ICE enhances adipogenesis. PPARγ and C/EBPα, master transcription factors for adipocyte differentiation and adipogenesis, were both significantly upregulated in epididymal fat of ICE mice (Fig. 5F), consistent with the notion that ICE enhances adipogenesis in WAT.

## Discussion

BAT-mediated thermogenesis is a calorie-consuming process that might be utilized to correct the energy surplus that underlies obesity in humans. Consistent with previous studies, our study indeed showed that ICE increases BAT recruitment. In addition, we found a significant reduction of body fat within hours of cold exposure in both our ACE and ICE protocols. Surprisingly, between successive rounds of cold exposure in the ICE protocol, we observed re-expansion of adiposity to a level beyond the basal level of the preceding cycle. This finding of a net increase in fat mass in our ICE mice is in line with a previous mouse study [19], but is at variance with several ICE rodent studies [17], [18], [20]–[22] and the two recent human studies, which either detected a reduction of body fat after 6 weeks of daily 2 h cold exposure [23] or observed no change in adiposity after 10 days of a similar ICE protocol (personal communication with Dr. Wouter D. Van Marken Lichtenbelt) [24]. The discordance is likely multifactorial and may include differences in species and experimental protocols. However, unlike these studies that measured body weight or single fat pad mass at the end of study, we used the highly accurate MRI approach to track body composition throughout the course of ICE treatment. We revealed dynamic changes of body fat during and after cold exposure. Our studies demonstrate that despite enhancing BAT recruitment and decreasing of fat mass during cold exposure, the overall consequence of ICE is fat accumulation in mice.

Another important finding of this study is that cold exposure profoundly impacts not only BAT but also liver and WAT, which might play a critical role in expanding adiposity between cold exposure periods. Under physiological conditions, cold exposure induces a series of responses from various organs to maintain body temperature, including BAT activation and TG mobilization. Consistent with previous reports [27], [30], our study showed that cold exposure dramatically increased HSL phosphorylation levels in WAT, indicative of increased lipolysis. Concomitantly, the lipogenic transcription factor SREBP1c was reduced in both WAT and liver. These results further confirm the notion that during cold exposure, energy consuming process of lipogenesis is suppressed while FA release from WAT is enhanced to fuel thermogenesis in BAT [15], [16]. The rapid decrease of fat mass during cold exposure further supports it. Interestingly, our study also demonstrated compensatory fat accumulation during the non-cold exposure period. The oscillation of fat mass during ICE is mirrored by the expression levels of key lipogenic genes in both WAT and liver. These results suggest a stimulatory effect of ICE on de novo lipogenesis during the non-exposure period. The liver is an important organ for lipid metabolism, where fatty acids are synthesized and/or reesterificated and incorporated into TG-enriched lipoproteins for transport to WAT and other tissues [31], [32]. We found both liver TG content and hepatic TG export were increased in ICE-treated mice. Together, these results indicate that in-between cold exposures, ICE mice increased lipogenesis in liver and WAT to offset cold-induced consumption of TG toward thermogenesis. This adaptive process manifested as increased fat mass of ICE mice.

Adipose tissue can expand through hypertrophy (increase in cell volume) and/or hyperplasia (increases in cell number). Our study showed that ICE increased the mass of both inguinal and epididymal fat. Consistent with previous reports [33]–[35], beige cells were detected mainly in the inguinal fat of ICE-treated mice. Apparently, beige cell recruitment only partially explains the ICE-induced fat tissue expansion. The reason for cold-induced subcutaneous fat specific browning is not clear. Although cold exposure activates SNS in almost all adipose depots, norepinephrine turnover rates were higher in inguinal fat than epididymal fat [36]. Since SNS plays a critical role in beige cells recruitment in WAT, higher norepinephrine turnover rates may contribute to the browning in inguinal fat. By analyzing white adipocyte cellular areas, we found that adipocytes of inguinal fat (beige cells were excluded) of ICE-treated mice were significant larger than that of control mice, while the opposite was true for epididymal fat (Fig. 4B). These results indicate WAT depot selectivity of ICE-induced metabolic adaptation. This finding is not surprising given the well-documented regional difference in adipocyte biology [37]. Subcutaneous adipocytes are less active in lipolysis than visceral adipocytes [38], [39] and do not undergo hyperplasia in response to cold exposure [40]. We therefore postulate lipolytic resistance, coupled with enhanced lipogenesis, results in lipid accumulation (hypertrophy) of inguinal adipocytes in ICE mice. In contrast, epididymal adipocytes were smaller than those in control mice, suggesting that the expansion of epididymal fat pad in ICE mice was primarily due to increased adipogenesis. This notion is further supported by the finding of significantly elevated adipogenic transcription factors C/EBPα and PPARγ. In addition, a recent study also demonstrated that even an overnight cold exposure induces adipocytes recruitment in epididymal fat [40]. We therefore propose that ICE-induced epididymal fat enlargement is mainly due to hyperplasia.

Energy balance plays a critical role in controlling fat accumulation. Cold exposure induces food intake in rodents. Our study indeed showed that ICE transiently increased food intake. However, our dietary intervention study showed that food restriction did not prevent ICE from causing expansion of adiposity, arguing against hyperphagia as the major cause of ICE-induced fat accumulation. On the other hand, previous studies have demonstrated that cold exposure increases energy expenditure in mice [22], [41], [42]. Due to the limitation of our facility, the energy expenditure of our mice was measure only at room temperature, where no significant changes were detected after 12 days of ICE. Nevertheless, it is unlikely that a decrease of energy expenditure during cold exposure contributed fat accumulation in ICE-treated mice. This raises the question of where the increased adiposity come from. Besides mild hyperphagia and de novo adipogenesis of beige cells and white adipocytes in subcutaneous fat, we also found a reduction of lean mass during cold exposure that recovered less robustly than fat mass during non-exposure periods (data not shown). Therefore, we hypothesize that ICE shifts the metabolism in favor of lipogenesis at the expense of muscle anabolism during non-exposure periods, which contributes to the fat accumulation. Although our study did not see any difference in energy expenditure, ICE treatment increased RER during light cycle and diminished the RER oscillation (Fig. 2J&K). This result indicates that ICE alters fuel source of metabolism and further supports the in favoring lipogenesis notion. However, further studies are required to verify this hypothesis.

WAT and BAT are distinct adipose tissues that are essential to energy storage and dissipation respectively. Despite playing opposite roles in energy expenditure, they act in concert to maintain energy homeostasis under physiological conditions. Our current study found that despite the induction of BAT recruitment and the reduction of WAT mass during cold exposure, the net effect of repetitive cold exposure is enhanced basal de novo lipogenesis and lipid accumulation in mice. Because fatty acids are the main fuel for non-shivering thermogenesis, increased lipid accumulation may help the mice cope with subsequent cold exposure. Therefore, we conclude that ICE increases body fat accumulation in mice by stimulating de novo lipogenesis during non-cold-exposure period.

## Supporting Information

Fig. S1 Cold exposure reduces body fat, but more body fat accumulated next day before cold exposure. Body composition was scanned before and after each round of cold exposure.
(TIF)

Fig. S2 Effect of ICE on liver histology. After 12 days of ICE treatment, liver tissues were collected and stained with hematoxylin and eosin.
(TIF)
