# Supplementary figures and images for: Intermittent Cold Exposure Enhances Fat Accumulation in Mice
Source: PLoS One. 2014 May 2;9(5):e96432. doi: 10.1371/journal.pone.0096432 (PMC4008632; doi:10.1371/journal.pone.0096432)

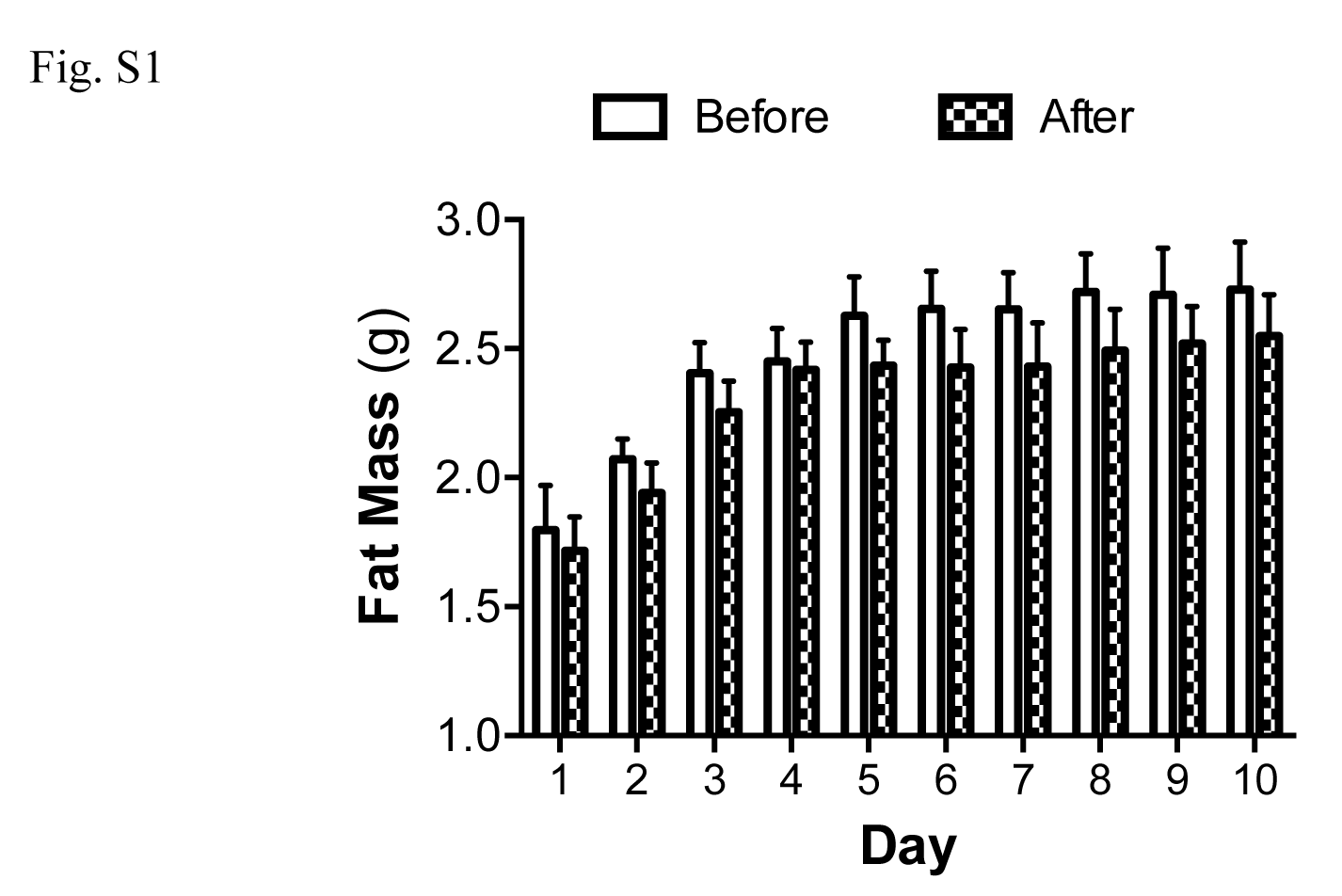

Supplement: Fig. S1 — Cold exposure reduces body fat, but more body fat accumulated next day before cold exposure. Body composition was scanned before and after each round of cold exposure. (TIF) [file pone.0096432.s001.tif]

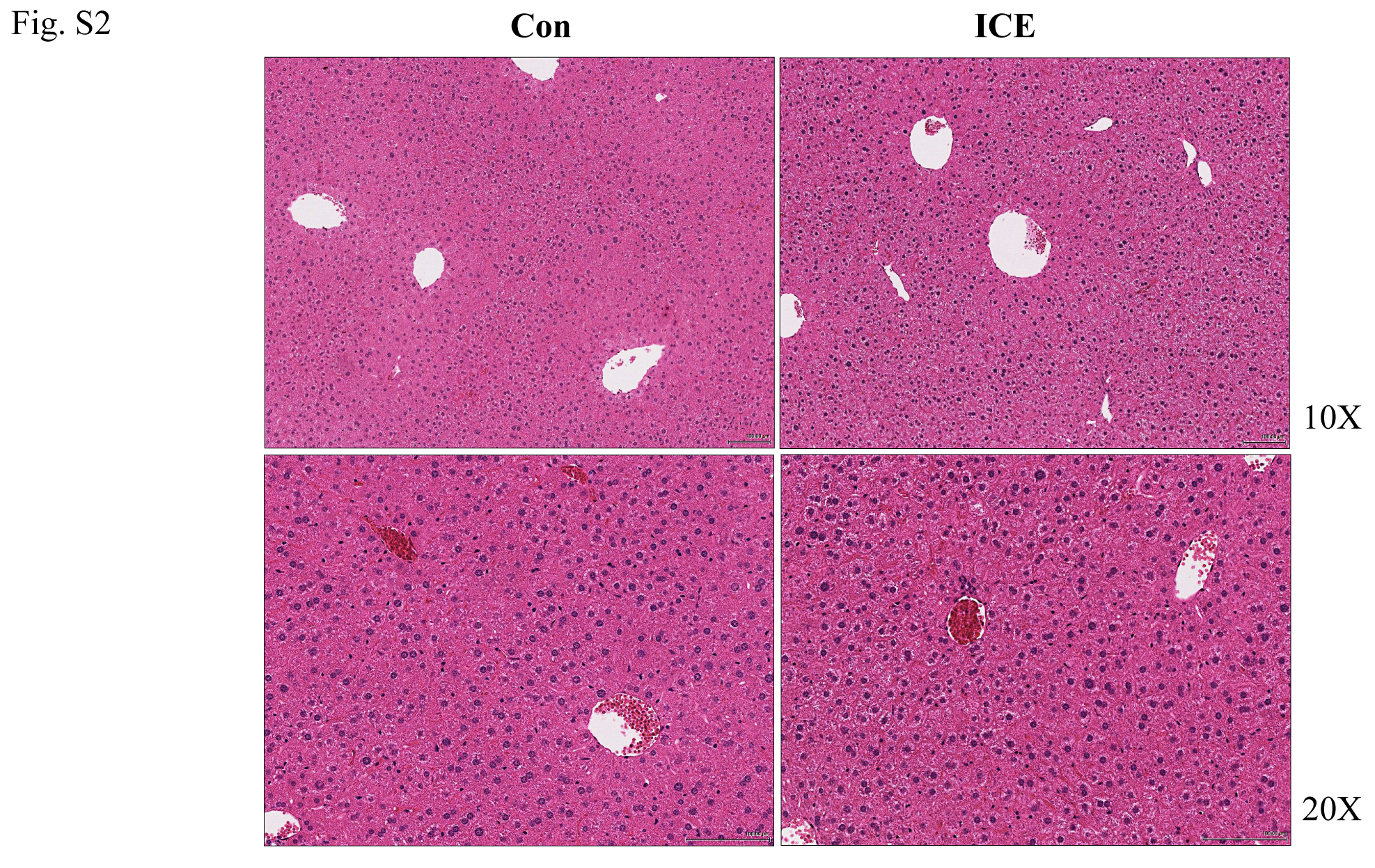

Supplement: Fig. S2 — Effect of ICE on liver histology. After 12 days of ICE treatment, liver tissues were collected and stained with hematoxylin and eosin. (TIF) [file pone.0096432.s002.tif]
